# Verification of False Reactivity in HIV Rapid Diagnostic Tests and Its Translation Into the Three Tests Algorithm in Cameroon

**DOI:** 10.1002/jcla.70333

**Published:** 2026-08-11

**Authors:** Rogers Ajeh Awoh, Désiré Takou, Isidore Oscar Bienvenu Etogo Ondigui, Roger Onana, Etienne Kembou, Collins Chenwi, Daniel Mabongo, Grace Beloumou Angong, Laetitia Bissai, Aude Christelle Ka'e, Alex Durand Nka, Ezechiel Ngoufack Jagni Semengue, Nadine Fainguem, Daniele Simnoue, Florine Tsimene, Sandrine Djupsa, Jaurès Metambou, Aurelie Kengne, Arlette Messeh, Gilbert Tchatchoua, Naomi‐Karell Etame, Michel Tommo, Krystel Zam Nnomo, Edith Temgoua, Basil Yaba, Gregory Edie Halle Ekane, Carlo‐Federico Perno, Vittorio Colizzi, Nicaise Ndembi, Diaby Ousmane, Joelle Bouba Pamen, Alexis Ndjolo, Celine Lastrucci, Joseph Fokam, Malachie Manouada

**Affiliations:** ^1^ Central Technical Group National AIDS Control Committee, Ministry of Public Health Yaoundé Cameroon; ^2^ Global Funds and Partners' Grants Coordination Unit Ministry of Public Health Yaoundé Cameroon; ^3^ Chantal BIYA International Reference Centre for Research on HIV/AIDS Prevention and Management Yaoundé Cameroon; ^4^ World Health Organization Country Office Yaoundé Cameroon; ^5^ Faculty of Medicine and Surgery University of Rome Tor Vergata Rome Italy; ^6^ Management Sciences for Health Country Office Yaoundé Cameroon; ^7^ Directorate of Pharmacy, Medicine and Laboratory Ministry of Public Health Yaoundé Cameroon; ^8^ Faculty of Health Sciences University of Buea Buea Cameroon; ^9^ Bambino Gesu Pediatric Hospital Rome Italy; ^10^ Faculty of Medicine Centre Hospitalo‐Universitaire Ndjamena Chad; ^11^ UNESCO Board of Biotechnology University of Rome Tor Vergata Rome Italy; ^12^ Africa Centres for Disease Control and Prevention Addis Ababa Ethiopia; ^13^ International Vaccine Institute Kigali Rwanda; ^14^ Directorate of Studies and Projects Ministry of Public Health Yaoundé Cameroon; ^15^ Faculty of Economics and Management Sciences University of Yaoundé II Soa Cameroon; ^16^ Directorate of Financial Resources and Heritage Ministry of Public Health Yaoundé Cameroon; ^17^ Faculty of Medicine and Biomedical Sciences University of Yaoundé I Yaoundé Cameroon; ^18^ World Health Organization Global HIV Hepatitis, and STI Programmes Geneva Switzerland; ^19^ Cabinet, Ministry of Public Health Yaoundé Cameroon

**Keywords:** antibody, Cameroon, false positive reactions, HIV Serodiagnosis, HIV seroprevalence, rapid diagnostic test

## Abstract

**Background:**

In a context of HIV seropositivity below 5%, transitioning from a two‐ to a three‐test algorithm is required to secure positive predictive values > 99% for mitigating false reactivity. With Cameroon having 2.7% seroprevalence, we sought to ascertain events of false reactivity from eight WHO‐prequalified HIV rapid diagnostic tests (RDTs) and design suitable combinations for the country three‐test algorithm.

**Methods:**

A facility‐based study was conducted in July–August 2021 among 172 consenting clients attending health facilities in Yaoundé‐Cameroon. Whole blood was collected and samples characterized in parallel using KHB Diagnostic Kit for HIV (1 + 2) Antibody and INNO‐LIA HIV I/II Ag/Ab to rule out reactive cases. Two lots of each selected RDT were tested simultaneously in quadruplicate on HIV non‐reactive samples and repeated by a different tester. RDTs were considered false positive if any reactivity was detected.

**Results:**

Following characterization, 25 samples were HIV‐reactive (i.e., ruled out) and 147 non‐reactive. Following random selection, 100 non‐reactive samples tested for false reactivity revealed: 0% (0/100) First Response HIV 1–2‐O Card, 0% (0/100) First Response HIV1 + 2/Syphilis Combo, 0% (0/100) STANDARD Q HIV/Syphilis Combo, 0% (0/99) ONE STEP Anti‐HIV1&2 (one invalid result); 1% (1/100) Determine HIV Early Detect and Bioline HIV/Syphilis Duo, 2% (2/100) Alere Determine HIV‐1/2, 4% (4/100) Rapid Test for Antibody to HIV.

**Conclusion:**

In the Cameroonian context, HIV false reactivity ranged from 0% to 4% across selected RDTs. Thus, a reliable three‐test algorithm ranks First Response HIV 1–2‐0 Card, First Response HIV1 + 2/Syphilis Combo or STANDARD Q as suitable RDT‐1 to mitigate false‐positive events at the country‐level.

## Graphical abstract

Verification study enabled the detection of HIV false reactivity ranging from 0‐4% across rapid diagnostic tests. A reliable three‐tests algorithm ranked First Response HIV 1‐2‐0 Card, First Response HIV1+2/Syphilis Combo or STANDARD Q as suitable test 1 to mitigate false positive events. As countries move toward HIV elimination, implementing the three‐tests algorithm based on tests with minimal (if any) cross‐reactivity is highly encouraged.

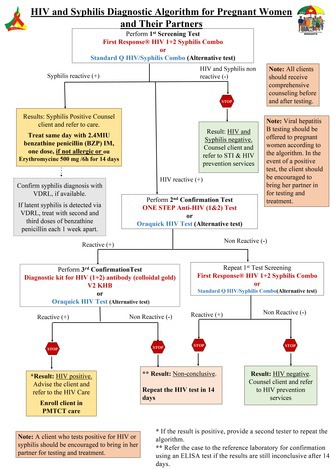

## 1 Introduction

At the global level, 40.8 million people were living with HIV as of 2024, and the majority of them are in low‐ and middle‐income countries (LMICs), and Sub‐Saharan Africa remains the most affected, accounting alone for 26.3 million people (64.5% of the global epidemic). In the West and Central Africa subregion, Cameroon is among the countries with the highest HIV prevalence (2.7%) with reference to 1.1% in the subregion [1]. The country has been experiencing a declining epidemic over the past ten years (50% reduction in prevalence and new infections), indicating success in prevention and treatment programs [1].

HIV diagnosis remains critical for initiating antiretroviral therapy (ART) or referring negative clients for prevention programs. In LMICs, HIV rapid diagnostic tests (RDTs) are widely used for screening based on their availability, affordability, user‐friendliness, rapid result delivery, and easy interpretation. RDTs often target HIV‐specific antibodies and/or antigens in blood, serum, or oral fluids, with a minimum sensitivity of 99% and a specificity of at least 98% as per World Health Organization (WHO) guidelines, which thereby ensures accuracy in detecting HIV while minimizing false positives [2].

In LMICs with low HIV prevalence or seropositivity (< 5%), the WHO recommends using a three‐test algorithm in order to minimize events of false positive. Thus, transitioning from a two‐ to a three‐tests algorithm requires a verification study to ensure that the new diagnostic algorithm is built on three different RDTs that sufficiently achieve the desirable positive predictive value (PVV) of > 99%, as per WHO recommendations for improved quality in the national HIV diagnostic strategy [3].

With a dropdown of HIV prevalence in Cameroon below 5% from the national health and demographic survey of 2011 (4.2%) [4], followed by a continuous declining trend to 2.7% prevalence from the national health and demographic survey of 2017 [5], the risks of HIV misdiagnosis due to false‐positive results raised concerns, and transitioning from the two‐ to a three‐tests algorithm became a priority for the national HIV testing program. Beyond WHO prequalification, the operational characteristics of WHO‐prequalified (PQ) diagnostic assays may altered by the testing analyte, type of specimen/sample, HIV clade diversity, target population, or geographical setting. This underscores the relevance of a verification study to guide the selection of WHO‐PQ RDTs for inclusion in the three‐test algorithm [6].

From a programmatic perspective, evidence from the verification would guide on designing the three‐test algorithm guided by core principles: (a) prioritize RDTs without or with low rate of false reactivity (< 2% at maximum); (b) each RDT in the testing algorithm should have a sensitivity of ≥ 99%, while RDT‐1 should have ≥ 98% specificity while RDT‐2 and RDT‐3 should have ≥ 99% specificity; (c) testing of specimens/samples should be done in a serial approach, meaning that the result of RDT‐1 is read and interpreted before deciding to proceed to RDT‐2, and so on; (d) RDT‐3 should not be used as a tiebreaker to rule in HIV infection, but RDT‐3 should rather be used to rule out HIV infection; (e) testing algorithms should be verified locally (i.e., national); (f) retesting of all people newly diagnosed HIV‐positive should be done using the nationally verified testing algorithm prior to ART initiation (https://gh.bmj.com/content/5/5/e001939).

With the goal to secure an acceptable positive predictive value in the national HIV testing algorithm in the frame of a low and declining HIV prevalence in Cameroon, we sought to ascertain events of false reactivity and shared reactivity from eight WHO‐prequalified HIV rapid diagnostic tests (RDTs) in order to design suitable combinations for the country three‐test algorithm with low rate of false positivity.

## 2 Methods

### 2.1 Study Design, Sites and Populations

A facility‐based study was conducted between July and August 2021 among consenting clients attending a routine clinic for HIV testing in four health facilities (*Yaoundé Central Hospital, Yaounde Jamot Hospital, Etoug Ebé Baptist Hospital, Biyem‐Assi District Hospital*) in the city of Yaoundé, Cameroon. Laboratory analyses for the study were conducted in the virology laboratory of the Chantal BIYA International Reference Centre for research on HIV/AIDS prevention and management (CIRCB, https://www.circb.cm/btc_circb/web/), a WHO designated national reference laboratory for HIV drug resistance surveillance (WHO ResNet), available at https://cdn.who.int/media/docs/default‐source/hq‐hiv‐hepatitis‐and‐stis‐library/list_of_who‐designated_hiv_drug_resistance_laboratories_oct‐2025.pdf?sfvrsn=c2b253ac_11.

### 2.2 Enrolment of Study Participants

Participants were clients attending HIV voluntary counseling and testing (VCT) services, antenatal care, PMTCT services, or key populations. Clients received the study information and provided informed consent for participation. Eligible clients were aged 20 years and above, provided informed consent, had a known negative or an unknown HIV status, were visiting the study site for voluntary HIV testing and provided whole blood samples as per the study protocol. Venous whole blood specimens were collected via venipuncture into EDTA tubes. Plasma was subsequently separated, aliquoted, stored at −80°C, and thawed once prior to testing. All characterization and verification analyses in this study were performed using plasma specimens under controlled laboratory conditions, in accordance with manufacturer's recommendations for the eight RDTs under evaluation.

### 2.3 Selection of Rapid Diagnostic Tests (RDTs)

Based on WHO prequalification criteria, eight RDTs were selected for this evaluation, taking into consideration their intrinsic performance (sensitivity and specificity); operational characteristics favoring implementation in LMICs; and the selected RDTs were in use or readily available in the health system, affordable, user‐friendly, and short turn‐around‐time (https://extranet.who.int/prequal/sites/default/files/document_files/231020_prequalified_IVD_product_list.pdf).

With the goal to meet‐up clinical expectations for both the general population and pregnant/breastfeeding women, the selection of RDTs for the verification study included five RDTs targeting only HIV1/2 (general population) and three dual HIV/syphilis RDTs (pregnant/breastfeeding women). Targeting dual HIV–syphilis in the latter population was driven by the increasing burdens of syphilis among pregnant/breastfeeding women in Cameroon, coupled with the low‐testing coverage of syphilis in antenatal care (ANC).

Operational criteria for RDTs selection were based on: (1) being listed in the WHO prequalification database, (2) suitable for use as point‐of‐care RDTs, (3) meeting WHO standards for sensitivity and specificity for HIV testing assay, (4) stability at room temperature ranging from +2°C to +30°C, (5) long shelf‐life post‐manufacturing, (6) short turnaround time for result delivery, (7) very low risk of inter‐reader variability in result interpretation, (8) affordable in term of cost.

### 2.4 Characterization of Clinical Samples

Characterization of each clinical sample collected from the study participants was performed at the CIRCB using a combination of two tests in a parallel approach: the KHB Diagnostic Kit for HIV (1 + 2) Antibody and the line immunoassay (LIA), INNO‐LIA HIV1/2 Ag/Ab assay. This parallel testing used for sample characterization served in ruling out samples with any sign of reactivity. Thus, both the KHB and INNO‐LIA assays were performed independently and in parallel (not sequential/serial) on all specimens to exclude any possible HIV reactive sample. Samples were classified as non‐reactive only if both characterization assays yielded negative results, thereby ensuring a high reliability of true‐negative panel for the verification of false reactivity. Consequently, clinical samples with a reactive or inconclusive result on any of the characterization assays were systematically ruled out from the study panel and excluded from the verification panel.

### 2.5 Procedure for the Verification of HIV False Reactivity

The verification of HIV false reactivity from the eight RDTs was done on a set of 100 clinical samples that were classified as non‐reactive after sample characterization. These 100 non‐reactive clinical samples were selected by an automated randomization, which provided equal selection among the non‐reactive samples, reduced risks of bias, and strengthened the quality of results to be reported for reliability and representability at population levels.

Testing each of the eight RDTs was done in two quadruplets (with different lot numbers for each RDT) and simultaneously (in parallel) by two trained laboratory testers as per the manufacturers' instructions; an independent result interpretation was done by each tester, and each result was confirmed by a second reader for a total of eight test results generated on each RDT and for each specimen/sample. Results interpreted independently by two readers for each RDT were then compiled for the overall analysis of false reactivity per RDT. In the event of a reactive or discordant result from any panel, a third reader was consulted before a final/consensus validation by the study investigative team. The two different lots used for each RDT therefore served to evaluate batch‐to‐batch consistency. As per standard recommendations for a verification study, any single reactive result observed on any replicate, irrespective of lot number, was conservatively classified as false reactivity for the corresponding RDT; similarly, any single invalid result observed during the testing process was classified as an invalid outcome for the RDT of interest.

### 2.6 Interpretation of False Reactive Results

At the end of the laboratory testing, samples were either classified as HIV‐reactive (indicating an RDT with false reactivity), non‐reactive (indicating an RDT with an accurate result of HIV seronegativity) or invalid (nonconclusive) result. The rate of false reactivity on any RDT was considered desirable performance if ≤ 2% versus poor performance if > 2%. Desirable performances were further classified as excellent (0% false reactive), moderate (1% false reactive), or fair (2% false reactive). Result validation was done by a technical working for transition to the HIV three test algorithm under decision N°1403/D/MINSANTE/SG/UCS‐FMP of the Ministry of Public Health (SDC1).

### 2.7 Ethical and Regulatory Considerations

The study was developed under the WHO guidance for assay verification [7] and carried out following the WHO standardized protocol for verifying reactivity (https://www.who.int/publications/i/item/9789240039162) [8]. Ethical clearance was provided by the National Ethics Committee for Research on Human Health (reference N^0^ 2021/02/1332); administrative authorization was issued by the Ministry of Public Health (reference N^0^ D19‐264). Each participant provided written informed consent prior to enrollment; data privacy of participants was ensured by the use of specific identifiers, while data confidentiality was duly considered by the use of the password encrypted computer for data storage and analysis.

## 3 Results

### 3.1 Study Participants and Sample Characterization

A total of 172 participants were enrolled in the study, with a median age: 29 [IQR: 23–38] years and 57% female including pregnant/breastfeeding women (*n* = 98); all appeared clinically asymptomatic and were classified as WHO clinical stage I.

Following sample characterization from the 172 specimens with the KHB and INNO‐LIA HIV1/2 Ag/Ac line Immunoassay (LIA), a total of 25 were found to be either reactive or inconclusive, giving an exclusion rate of 14.5%; the remaining samples (*n* = 147) non‐reactive for HIV were classified as HIV negative and eligible for the verification study. Of the eligible HIV‐negative samples, the automated random selection provided 100 samples (69% of eligibility samples) used for the verification of false reactivity from the eight WHO‐PQ RDTs.

### 3.2 Description of RDTs Under Verification

The eight WHO‐PQ RDTs under evaluation were: One Step Anti‐HIV (1&2) Test, Rapid Test for Antibody to HIV (Colloidal Gold Device), Alere Determine HIV‐1/2, Determine HIV Early Detect, First Response HIV 1–2‐0 Card Test, Bioline HIV/Syphilis Duo, First Response HIV1 + 2/Syphilis Combo Card Test, and Standard Q HIV/Syphilis Combo Test. Table **1** provides the detailed characteristics of these RDTs.

**TABLE 1:** Characteristics of selected RDTs.

N^o^	Product name	Clinical Sensitivity (%)	Clinical Specificity (%)	Shelf‐life upon Manufacture	Storage Temperature (°C)	Cost per Test
RDT1	One Step Anti‐HIV (1&2)	100.0	100.0	24	2–30	US$0.65
RDT2	Rapid Test for Antibody to HIV	100.0	98.5	18	2–30	US$0.70
RDT3	Alere Determine HIV‐1/2	100.0	98.9	18	2–30	US$0.90
RDT4	Determine HIV Early Detect	100.0	99.4	18	2–30	US$0.95
RDT5	First Response HIV 1–2‐0 Card	100.0	100.0	24	4–30	US$0.75
RDT6	Bioline HIV/Syphilis Duo	100.0	99.5	24	1–30	US$1.30
RDT7	First Response HIV1 + 2/Syphilis Combo Card	100.0	99.5	24	4–30	US$1.15
RDT8	Standard Q HIV/Syphilis Combo	100.0	99.5	24	2–40	US$0.95

Abbreviations: %, percentage; °C, degree centigrade; RDT, rapid diagnostic test.

### 3.3 Verification of RDTs With False Reactivity

Among non‐reactive specimens included in the verification study, only One Step Anti‐HIV (1&2) Test reported an invalid result on one sample, giving a total 99 conclusive results. All other seven RDTs showed conclusive results on all 100 specimens and their respective replicates. False reactive rates varied from 0% to 4%, with 7/8 (87.5%) RDTs achieving a desirable performance (Table 2). In order of performance, RDTs were classified as:

- Excellent performance (0% false reactivity): two HIV and two HIV/Syphilis RDTs;
- Moderate performance (1% false reactivity): one HIV and one HIV/Syphilis RDT;
- Fair performance (2% false reactivity): one HIV RDT;
- Poor performance (4% false reactivity): one HIV RDT

**TABLE 2:** Detailed performance of the RDTs following verification analyses.

N^o^	Product name	Invalid result (%)	False reactivity % (*n/N*)	Performance (%)	Ranking in algorithm
RDT1	One Step Anti‐HIV (1&2)	1	0 (0/99)	Excellent	Preferred Test 2
RDT2	Rapid Test for Antibody to HIV	0	4 (4/100)	Poor	Not appropriate
RDT3	Alere Determine HIV‐1/2	0	2 (2/100)	Fair	Least possible Test 1
RDT4	Determine HIV Early Detect	0	1 (1/100)	Moderate	Possible Test 1
RDT5	First Response HIV 1–2‐0 Card	0	0 (0/100)	Excellent	Preferred Test 1
RDT6	Bioline HIV/Syphilis Duo	0	1 (1/100)	Fair	Possible Test 1
RDT7	First Response HIV1 + 2/Syphilis Combo Card	0	0 (0/100)	Excellent	Preferred Test 1
RDT8	Standard Q HIV/Syphilis Combo	0	0 (0/100)	Excellent	Preferred Test 1

Abbreviations: %, percentage; RDT, rapid diagnostic test.

Regarding shared false reactivity, only one (1%) sample yielded false reactive results on both the Bioline HIV/Syphilis Duo (RDT6) and the Rapid Test for Antibody to HIV (RDT2). Thought the molecular basis (e.g., shared antigenic components or nonspecific binding) was not investigated in this study, our finding indicates an event of shared false reactivity event between these two RDTs on the same clinical sample (Table 3).

**TABLE 3:** Level of false reactivity and shared false reactivity among RDTs.

	RDT1	RDT2	RDT3	RDT4	RDT5	RDT6	RDT7	RDT8
RDT1	0%							
RDT2	0%	3%						
RDT3	0%	0%	2%					
RDT4	0%	0%	0%	1%				
RDT5	0%	0%	0%	0%	0%			
RDT6	0%	1%	0%	0%	0%	1%		
RDT7	0%	0%	0%	0%	0%	0%	0%	
RDT8	0%	0%	0%	0%	0%	0%	0%	0%

Abbreviation: RDT, rapid diagnostic test.

**FIGURE 1:**
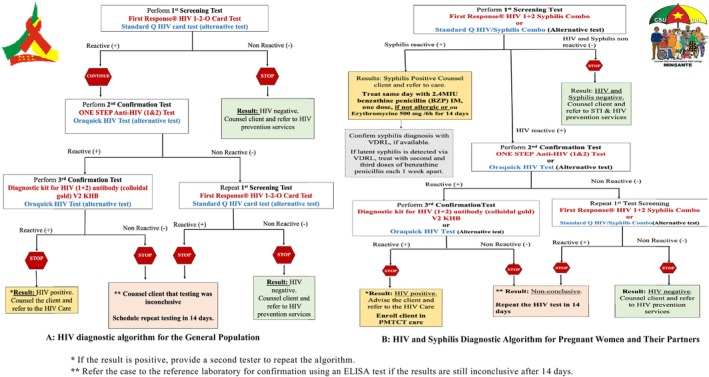
HIV Three‐Test Algorithm for the general population and pregnant/breastfeeding women.

Based on the false reactivity rates of the RDTs, a preferred RDT combination to build the national three tests algorithm was the First Response HIV 1–2‐0 Card Test as preferred RDT‐1 for the general population, and First Response HIV1 + 2/Syphilis Combo as preferred RDT‐1 for the population of pregnant/breastfeeding women, their partners and key populations. As preferred RDT‐2, ONE STEP Anti‐HIV (1&2) Test was the most suitable. Taking into consideration false reactivity of some RDTs, the selection of alternative RDT‐2 and RDT‐3 (Oraquick on whole blood, serum or plasma, but not self‐test) was based on their satisfactory performance and operational characteristics as assays known to be highly specific for confirmation of any HIV infection (with a consensus validation by the technical working group). Figure 1 provides preferred and alternative RDTs for use in the HIV three‐test Algorithm.

## 4 Discussion

The goal of the present study aimed at verifying events of false reactivity occurring with RDTs as the country transitions from a two‐ to a three‐test algorithm, in order to strengthen the HIV testing program with the frame of a low seropositivity in Cameroon. The use of a three‐test combination with minimal false positivity will therefore contribute to limiting issuance of false positive results that can be encountered with the two tests diagnostic strategy, especially with the low HIV prevalence in Cameroon (2.7%) [1].

Among the tested RDTs, First Response HIV 1–2‐0 Card Test, First Response HIV1 + 2/Syphilis Combo Card Test, Standard Q HIV/Syphilis Combo Test, and One Step Anti‐HIV (1&2) Test demonstrated no false reactivity. These findings are consistent with the manufacturers' reported characteristics, which indicate a specificity of 100%. This result is consistent with the findings of Boadu et al., who observed a 100% specificity when whole blood and plasma specimens were used with the First Response HIV 1–2‐0 Card Test. However, their study also reported a possible decrease in specificity when serum samples were tested in 2015 [6], in contrast to the excellent performance reported with the current version of the RDT (see Table 1). In contrast, a 2018 laboratory evaluation conducted in Peru reported a specificity of 99.5% for HIV antibody detection using the Standard Q HIV/Syphilis Combo test, whereas our study demonstrated no false reactivity for the same test [8]. Underscoring the need for rigorous quality control, particularly in decentralized settings, where rapid tests are widely used, including in community‐based campaigns.

The Rapid Test for Antibody to HIV (Colloidal Gold Device) exhibited the highest false reactivity rate (4%), followed by the Alere Determine HIV‐1/2 (2%), and both the Determine HIV Early Detect and Bioline HIV/Syphilis Duo tests (1% each). These rates of false reactivity, particularly for the Rapid Test for Antibody to HIV, remain concerning in low‐prevalence settings. Including such tests in diagnostic algorithms can significantly reduce the positive predictive value, potentially leading to misdiagnosis, unnecessary psychological stress, additional confirmatory testing, and even inappropriate treatment of individuals who are not truly infected. A systematic review and meta‐analysis of studies published between January 2012 and December 2016, which evaluated the performance and operational characteristics of dual tests for HIV and syphilis, reported that the specificity values for HIV diagnosis using the SD Bioline HIV/Syphilis Duo Test ranged from 97% to 100%, indicating a false reactivity rate between 0% and 3% [9]. A multicenter study conducted across six sites in Sub‐Saharan Africa reported a false reactivity rate of 8.4% for Alere Determine HIV‐1/2 and 2.9% for SD Bioline. These results indicate a higher proportion of false reactivity compared to our study [10]. The same study reported a 7.3% shared false reactivity between Alere Determine HIV‐1/2 and SD Bioline, a pattern that was not observed in our study. However, a notable finding of our study was the shared false reactivity between the SD Bioline HIV/Syphilis Duo and the Rapid Test for Antibody to HIV, where one sample tested falsely reactive on both assays. This shared false positivity may suggest shared antigenic components or similar assay chemistries leading to nonspecific binding.

The dual HIV/syphilis RDTs, particularly the STANDARD Q and First Response Combo, demonstrated excellent performance (no false reactivity), hence supporting its use as alternative assay in our context for integrated HIV/syphilis testing within maternal health services. Such integration is particularly relevant in the context of triple elimination activities supported by the Global Fund, PEPFAR and the government of Cameroon [11]. In addition to the known intrinsic performance of RDTs, LMICs are therefore encouraged to adapt the three tests algorithm in their context based on locally‐generated findings for efficiency [12].

The success of the adopted three‐tests algorithm relies on the development of a transition plan to ensure an effective implementation of the new algorithm. This transition plan in Cameroon has a one year timeline and used for monitoring and evaluation (https://cdnss.minsante.cm/sites/default/files/Plan%20Algorithme%20aĚ%203%20tests%2014‐06‐2024.pdf). This approach ensures a robust quality management system for reliability of HIV result delivery in all testing sites, accurate data recording and reporting, efficiency in supervision for quality assurance with standard registers, and timely corrective actions whenever need be to mitigate misdiagnosis in the frame of a declining epidemic [13].

Shared false reactivity of RDTs is not unique to the Cameroonian context. Several countries enrolled in this transition process have also reported similar findings, most of which guided toward the revision of RDTs for use in their preferred three‐tests algorithms, both in African and European countries [14].

Importantly, while the First Response HIV 1–2‐0 Card Test demonstrated 100% no cross‐reactivity in our study, previous work has shown that specificity might drop to 83% when serum samples are used [15], thereby underscoring the preferential use of whole blood or plasma specimens. More importantly, our recent study on HIV‐1 group O strains from Cameroon demonstrated that transitioning from the previous two‐test to the newly established three‐test algorithm has improved the national program diagnostic performance on currently circulating HIV‐1 group O in the country [16]. This additional evidence further supports and validates the developed three‐test algorithm for routine use in the Cameroonian context.

A study constraint was the limited number of RDTs (eight in total) during our study (as per funding availability). Even though other verification studies had between 4 and 13 RDTs [14], the maximum number of RDTs as possible would give a wider range of alternative or options in building the most optimal testing algorithm. Henceforth, countries are encouraged to include a maximum number of RDTs during similar verification studies. Despite the lack of formal statistical analysis of inter‐assay and inter‐reader variability, any event of reactivity or invalid result was confirmed by two readers, following by a third reader before submission to a consensus approval done by the study team (highest level of validation). This approach therefore mitigates risk of misinterpretation and thereby strengthens findings from this study.

## 5 Conclusion

In the Cameroonian context, false reactivity events are observed with some RDTs, ranging from 0% to 4%, with limited shared (1% only) reactive across assays. Thus, transitioning to the three tests algorithm should prioritize RDTs without false reactivity, of which First Response HIV 1–2‐0 Card Test, First Response HIV1 + 2/Syphilis Combo, or STANDARD Q as RDT‐1. Such evidence‐based decisions would strengthen efforts toward HIV elimination in the frame of a declining epidemic at country‐ and global‐levels. Successful implementation requires a transition plan with clear milestones and quality management system.

## Author Contributions

Initiated the manuscript: R.A.A., D.T., I.O.B.E.O., J.F., and M.M. Revised substantially the manuscript: R.O., E.K., C.C., G.B.A., L.B., A.C.K., A.D.N., E.N.J.S., N.F., S.D., J.M., A.K., A.M., N.‐K.E., M.T., K.Z.N., E.T., D.O., J.B.P., A.N., C.L., D.M., D.S., F.T., G.T., B.Y., G.E.H.E., C.‐F.P., V.C., N.N. Designed the study or contributed in data collection: R.A.A., D.T., I.O.B.E.O., R.O., E.K., C.C., G.B.A., L.B., A.C.K., A.D.K., E.N.J.S., N.F., S.D., J.M., A.K., A.M., N.‐K.E., M.T., K.Z.N., E.T., D.O., J.B.P., A.N., C.L., J.F., M.M. Interpreted the data: D.M., D.S., F.T., G.T., B.Y., G.E.H.E., C.‐F.P., V.C., and N.N. Approved the final version of the manuscript: All coauthors.

## Funding

This work was supported by World Health Organization.

## Data Availability

The data that supports the findings of this study are available in the supporting information of this article.
